# Farmerfish gardens help buffer stony corals against marine heat waves

**DOI:** 10.1371/journal.pone.0282572

**Published:** 2023-03-08

**Authors:** Randi N. Honeycutt, Sally J. Holbrook, Andrew J. Brooks, Russell J. Schmitt

**Affiliations:** 1 Coastal Research Center, Marine Science Institute, University of California Santa Barbara, Santa Barbara, California, United States of America; 2 Department of Ecology, Evolution and Marine Biology, University of California Santa Barbara, Santa Barbara, California, United States of America; Helmholtz-Zentrum fur Ozeanforschung Kiel, GERMANY

## Abstract

With marine heat waves increasing in intensity and frequency due to climate change, it is important to understand how thermal disturbances will alter coral reef ecosystems since stony corals are highly susceptible to mortality from thermally-induced, mass bleaching events. In Moorea, French Polynesia, we evaluated the response and fate of coral following a major thermal stress event in 2019 that caused a substantial amount of branching coral (predominantly *Pocillopora*) to bleach and die. We investigated whether *Pocillopora* colonies that occurred within territorial gardens protected by the farmerfish *Stegastes nigricans* were less susceptible to or survived bleaching better than *Pocillopora* on adjacent, undefended substrate. Bleaching prevalence (proportion of the sampled colonies affected) and severity (proportion of a colony’s tissue that bleached), which were quantified for >1,100 colonies shortly after they bleached, did not differ between colonies within or outside of defended gardens. By contrast, the fates of 399 focal colonies followed for one year revealed that a bleached coral within a garden was a third less likely to suffer complete colony death and about twice as likely to recover to its pre-bleaching cover of living tissue compared to *Pocillopora* outside of a farmerfish garden. Our findings indicate that while residing in a farmerfish garden may not reduce the bleaching susceptibility of a coral to thermal stress, it does help buffer a bleached coral against severe outcomes. This oasis effect of farmerfish gardens, where survival and recovery of thermally-damaged corals are enhanced, is another mechanism that helps explain why large *Pocillopora* colonies are disproportionately more abundant in farmerfish territories than elsewhere in the lagoons of Moorea, despite gardens being relatively uncommon. As such, some farmerfishes may have an increasingly important role in maintaining the resilience of branching corals as the frequency and intensity of marine heat waves continue to increase.

## Introduction

Climate change is intensifying disturbances such as marine heat waves that increasingly are impacting shallow ocean ecosystems [1], including coral reefs [2, 3]. Marine heat waves (MHWs) can trigger a coral ‘bleaching’ event where thermal stress breaks down the mutualistic relationship between a coral host and its endosymbiotic algae (Symbiodiniaceae), which are expelled from the host. A bleached coral will die if it does not regain its endosymbionts. During the past several decades, episodes of mass mortality of corals from MHWs have contributed to the widespread decline of stony corals [3, 4], which typically has profound effects on many structural and functional aspects of local reef communities [5, 6]. There is ample evidence that the frequency, severity, and geographical extent of coral bleaching induced by MHWs have increased since the first such reported mass coral mortality event in the early 1980’s [5, 7–9]. Hence, understanding the abiotic and biotic factors that influence the ability of stony corals to buffer the effects of MHWs is critical for developing management strategies that will enhance the resilience of coral to recurring periods of thermal stress [3, 10–12].

Stony coral in parts of the Indo-Pacific can benefit by local interactions with a farming damselfish, the Dusky Gregory *Stegastes nigricans* (‘farmerfish’), that can result in enhanced recruitment, growth, and survival of coral colonies [10, 13–17]. *S*. *nigricans* is a group-living damselfish that occurs on tropical reefs from the western Indian Ocean to the eastern Pacific [18]. Territorial groups of this fish cultivate algal gardens for food that they vigorously defend against herbivores as well as corallivorous fishes [15, 19]. By protecting their gardens, farmerfish provide coral that recruit into the garden an associational defense against coral predators, which can enhance coral growth and survival and ultimately can result in a great number and diversity of corals within a garden compared to adjacent areas that are not defended by farmerfish [10, 13, 14, 17]. In addition to the moderating influence of *S*. *nigricans* on top-down effects on coral within their territory, farmerfish also can potentially strengthen bottom-up forcing via local fertilization of coral. Several experimental studies have revealed that group-living damselfishes can benefit stony corals and anthozoans by excreting nitrogenous waste that enhance their growth rate and/or reduce their probability of dying [20–26]. What has not been explored is whether coral-farmerfish interactions might buffer coral against MHWs, or alternatively, exacerbate the thermal stress to increase a coral’s probability of bleaching and not recovering.

There are *a priori* reasons to hypothesize that coral-farmerfish interactions could either benefit or harm coral colonies during a MHW. For example, the reduction in photosynthetically supplied energy a coral colony suffers due to fewer endosymbionts during a MHW might be offset to some degree by a reduced need to allocate energy to wound repair because farmerfish lessen corallivory [15]. Similarly, recycled nitrogen excreted by farmerfish that enhance algal productivity within their territories [27, 28] also might fuel a rapid increase in the density of the algal endosymbionts a bleached coral needs, and thus lower the probability it would starve to death [24, 25]. By contrast, there also are situations where too much nitrogen can stimulate an excess production of photosynthetic products that can increase the susceptibility to or severity of coral bleaching [11, 29]. Both empirical and theoretical studies that have examined nutrient enrichment in relation to bleaching severity suggest that the relationship can switch from beneficial at lower levels to harmful at higher levels of enrichment [11, 25, 29].

The aim of this study was to evaluate whether the response and fate of stony coral to a major thermal stress event depended on whether the colony occurred within a territorial garden protected by *Stegastes* or on adjacent, undefended reef substrate. At our study location (Moorea, French Polynesia), *Stegastes nigricans* live in shallow lagoon habitats. In early 2019, a prolonged marine heat wave in Moorea kept sea surface temperatures well above average for several months, which caused a substantial fraction of branching corals (mostly in the genera *Pocillopora* and *Acropora*) to bleach [4]. The present study focused on *Pocillopora* spp. because they have been the dominant branching coral on reefs of Moorea for the past several decades [30], and they commonly occur within farmerfish territories [10]. The responses (i.e., bleaching prevalence, bleaching severity) of *Pocillopora* colonies within and outside of farmerfish gardens on haphazardly selected lagoon patch reefs (hereafter bommies) were evaluated immediately after coral had bleached but before the bleached tissue had died. The fate of colonies that bleached (i.e., proportion that completely died, proportion that recovered) within and outside of farmerfish gardens was assessed by following 399 individually marked colonies for a year. Our findings indicate that gardens defended by *Stegastes* may serve as a thermal stress oasis for branching coral that enhances their chances of buffering marine heat waves.

## Materials and methods

### Study site, study organisms, and the thermal stress event

Moorea, French Polynesia (17° 30’S, 149° 50’W) is a high volcanic island in the central South Pacific Ocean ~ 20 km west of Tahiti. Moorea is protected by a barrier reef approximately 1 km offshore that encircles the ~ 60 km perimeter of the island, which creates a continuous series of shallow (~ 3 m depth) lagoons. Shoreward of the barrier reef crest are numerous patch reefs (‘bommies’) separated by sand, coral rubble, and reef pavement. Dispersed throughout the lagoons are colonies of the group-living Dusky Gregory (*Stegastes nigricans*), which tend to be most common from the mid-lagoon to fringing reef in the cross-shore direction [10, 31]. *Stegastes* farm gardens of turf algae and defend them against intruders [15, 32–37]. In Moorea, farmerfish territories are mainly established on dead portions of mounding coral bommies (*Porites* spp.) and reef pavement [10, 14], but also within thickets of living staghorn coral *Acropora pulchra* [15].

In 2019, reefs of Moorea were subjected to the largest thermal stress event in at least the previous 14 years [4]. Over the Austral summer of 2019, an unusually warm water event resulted in an accumulative thermal stress (~ 6 weeks above 29° C, the temperature threshold that predicts thermal stress for corals in Moorea) that was roughly twice that recorded in 2007, which was the highest recorded in any of the 14 years preceding the MHW of 2019. The 2019 thermal event peaked in April—May, at which time a substantial proportion of (mostly branching) coral in the lagoon and shallower (< 12 m) depths on the outer fore reef bleached [4, 30]. The present study was initiated in June 2019, after corals had bleached but before bleached tissues died, and continued until August 2020 to determine the fate of corals that had bleached. The study site was located over a 2 km longshore stretch of the lagoon at the eastern-most corner of the north shore of Moorea, between Avaroa Pass and Irihonu Pass; in the cross-shore direction, habitats were surveyed between the fringing reef to the mid-lagoon region, which is where *S*. *nigricans* gardens are abundant. The study focused on branching coral in the genus *Pocillopora* because they were by far the most common type of branching coral found in Moorea [30], including in *Stegastes* gardens [10].

### Resistance to bleaching: Patterns of prevalence and severity

A field survey to assess whether patterns of bleaching of *Pocillopora* differed between colonies within a farmerfish garden (territory) and those on adjacent, unprotected substrate was conducted during June and July 2019 before bleached tissues of coral died or recovered. Divers assessed all *Pocillopora* colonies found on 178 haphazardly encountered bommies that were scattered throughout the 2 x 0.5 km study polygon. The bommies varied in the amount of their surface covered by *Stegastes* gardens, from lacking gardens altogether to those that were partially to completely covered. Gardens were readily identified by their lush growth of turf red algae (commonly *Polysiphonia* spp.) and the presence of *Stegastes*; the substrate surface not defended by farmerfish typically had closely cropped turf and/or crustose coralline algae. All surveyed bommies were measured (L x W x H) and the location of *Pocillopora* colonies noted (outside or inside of a garden if present). Colonies were then placed into 1 of 5 diameter categories (< 3 cm, 3–10 cm, 11–20 cm, 21–30 cm, > 30 cm dia; see [11]). Each *Pocillopora* was photographed, and the proportion of the colony that was (1) alive and unbleached, (2) alive but bleached (i.e., tissues transparent and underlying skeleton visibly white), and (3) dead (no tissue and skeleton overgrown with algae from previous partial mortality) was estimated visually in the field (the 3 categories sum to 1). The live but bleached category was defined as tissue that had substantially lower or no pigmentation (due to lower levels of Symbiodiniaceae) relative to the rest of the colony and followed the Australian Coral Watch Coral Health Chart for scoring coral bleaching (https://coralwatch.org). Quantification of bleaching was made by the same individual (RNH). If present on a bommie, *Stegastes* garden sizes were measured, and the number of adult farmerfish enumerated. A total of 1,137 *Pocillopora* colonies was assessed on the 178 bommies surveyed.

Due to the need for a rapid assessment of bleached corals, the total amount of the two ‘substrate types’ (inside vs outside farmerfish territories) in the study area was not estimated during the June to July 2019 survey. Not all the non-garden area of a bommie was suitable for occupancy by *Pocillopora* since varying portions of the *Porites* coral bommies were still alive and/or occupied by other benthic space holders such as macroalgae. We used a recent survey of benthic habitats of the study site to estimate the relative areas of garden vs non-garden habitats that were suitable for occupation by *Pocillopora* as an approximation of the relative sampling effort for the two habitat types. The 2016 benthic survey consisted of 24 band transects each 20 x 5 m where the substrate / space holder was identified at every 0.5 m point on the grid (i.e., 492 point samples per band transect). The benthic categories quantified included, among others, *Stegastes* gardens, other turf algae, crustose coralline algae (CCA), live coral (by genus or species), live portions of *Porites* bommies, and macroalgae (by genus or species). The 24 transects were distributed more or less evenly from the mid-lagoon to fringing reef along the 2 km span of the study site. These data were used to estimate the mean proportion of hard substrate at the study site that was covered by farmerfish gardens, and the mean proportion of non-garden hard substrate that was or could have been occupied by branching corals (i.e., cover of branching coral + cover of cropped turf algae / CCA on bommies).

In addition to exploring how *Pocillopora* responded to the bleaching event as a function of position within or outside a farmerfish territory, responses as a function of colony size also were explored as other studies in Moorea have shown that for a variety of reasons, larger and smaller colonies of *Pocillopora* can have different susceptibilities to bleaching [4, 30]. Accordingly, the 1,137 surveyed corals were divided into two size classes, small (≤ 10 cm dia) and large (> 10 cm dia). Several statistical tests were conducted to explore relationships between *Pocillopora* colony size and location (inside or outside of a garden) with two metrics of bleaching (prevalence and severity, see below). All analyses were performed using the R language for statistical computing version 4.1.1 [38].

### Proportion of large vs small colonies as a function of garden status

A Pearson’s chi-square contingency analysis with Yates’ continuity correction was used to test whether the proportion of large vs small *Pocillopora* colonies differed between those found within and outside farmerfish gardens. For each garden ‘status’ (inside or outside), the number of colonies in a size class (large or small) was divided by the total number of colonies in that location category.

### Bleaching prevalence

Bleaching prevalence is the proportion of the total number of colonies in a sample that had bleached. For this analysis, a colony was categorized as bleached if ≥ 5% of its live tissue was deemed to be bleached using the criteria described earlier. To determine whether bleaching prevalence differed as a function of garden status (inside or outside) and colony size, Pearson’s chi-square contingency analyses with Yates’ continuity correction were used to test for garden status effect separately for small and large size classes. For each size class and garden status, the number of colonies categorized as bleached was divided by the total number of colonies (bleached + unbleached) in the respective size and garden status group.

### Bleaching severity

Bleaching severity is the proportion of the tissue on an individual colony that bleached. A two-way analysis of variance tested the effect of colony size (small or large) or location (inside or outside a garden) on the severity of bleaching. Data were arcsine-transformed to meet assumptions of normality; the interaction term in the full model was non-significant *(p =* 0.24*)*, so the analysis was rerun without the interaction term.

### Fate of bleached coral: Patterns of mortality and recovery

Bleached tissue on a coral will eventually die if it does not recover a sufficient complement of algal symbionts. To assess the post-bleaching fate of *Pocillopora* at the study site, focal colonies were selected and followed for a year. In July 2019, 399 *Pocillopora* colonies that had some level of bleaching (5–100%) were selected and uniquely tagged using combinations of colored cable ties that were attached to u-nails affixed to the adjacent substrate. The focal colonies, which were selected haphazardly (spread among 148 bommies), included 263 that occurred within farmerfish gardens and 136 that were on bommie surfaces outside of *Stegastes* gardens. Colonies within and outside of gardens were not formally paired, but were intermingled throughout the study site; in most cases, colonies outside of *Stegastes* gardens were within 1–2 m of those in gardens. Not all bommies had corals both inside and outside of a *Stegastes* garden. Colonies were resampled after 6 months (January 2020) and again after 13 months (August 2020), at which times the proportion of each colony that was alive or had died was quantified.

### Complete colony death

A subset of the focal corals suffered complete colony mortality between July 2019 and August 2020. Pearson’s chi-square contingency analyses with Yates’ continuity correction were used to test whether the proportion of corals that suffered complete colony mortality differed depending on whether they were inside or outside of a garden. The total number of dead colonies both inside and outside of gardens was divided by the total number of colonies in those categories. To explore whether smaller and larger sizes had different fates depending on their garden status, a Fischer’s exact test was used to test if colony size and garden status influenced whether a colony had complete mortality. The number of colonies within each garden status and size class was divided by the total number of colonies within those respective categories.

### Colony recovery

We explored if garden status influenced whether the amount of living tissue on colonies after a year was equal to or greater than the initial amount in 2019, immediately after they bleached. Corals that met this criterion were operationally considered as recovered. Pearson’s chi-square contingency analyses with Yates’ continuity correction were used to test if the proportion of colonies that recovered depended on whether they were inside or outside of a garden. The total number of colonies that recovered within a respective garden status was divided by the total number of colonies in that category. A Fischer’s exact test was used to test whether recovery of large and small corals differed depending on garden status. The number of colonies within each garden status and size category (large vs small) was divided by the total number of colonies within those respective categories.

## Results

### Patterns of *Pocillopora* distribution

**Fig 1:**
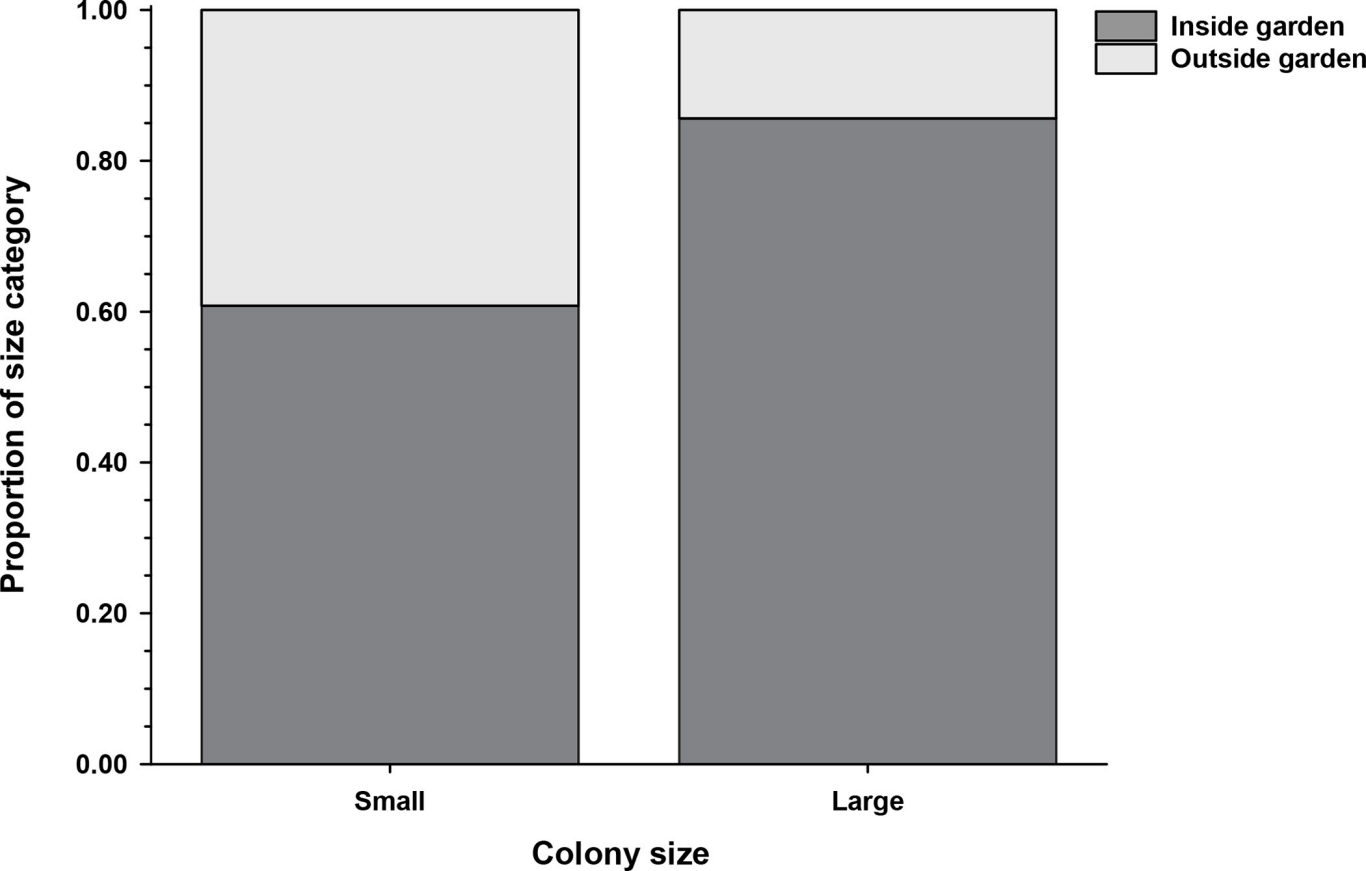
Proportion of small and of large *Pocillopora* as a function of farmerfish garden status. The proportion of small (≤ 10 cm dia) and of large (> 10 cm) *Pocillopora* colonies encountered inside and outside of farmerfish gardens of the 1,137 colonies surveyed on 178 bommies (small: N = 484 inside, 312 outside; large: N = 292 inside, 49 outside). The proportion of the two size classes inside vs outside of gardens was significantly different (*p* < 0.001).

Our estimates of benthic microhabitats in 2016 reveal that in the years just before the bleaching event, the mean proportion of bommie surfaces covered by *Stegastes* gardens was 0.30 (± 0.4 SE) within the study site, whereas the mean proportion of bommie surfaces that either were occupied by or suitable for branching corals was 0.49 (± 0.4); Blanchette et al. [39] reported similar values in a more recent survey. This suggests that in our 2019 survey of bleached coral on bommies, the suitable area sampled for *Pocillopora* outside of farmerfish gardens may have been as much as 1.6 times greater than the total area of farmerfish gardens sampled. Despite this likely sampling bias toward more non-garden habitat, only 32% of the *Pocillopora* colonies encountered (361 of 1,137) in the census of the 178 bommies were found outside of farmerfish territories. Perhaps even more striking, there was a marked shift in proportional representation of small versus large colonies between garden and non-garden habitats (Fig 1). About 60% of all smaller *Pocillopora* encountered in the survey occurred within gardens, which rose to 86% of the larger colonies (Fig 1). This shift between the size classes was highly significant (*X*^2^_(1, 1137)_ = 66.76, *p* < 0.001) and is a pattern consistent with enhanced survivorship and/or growth of coral within *Stegastes* territories relative to colonies on adjacent, non-garden substrates.

### Resistance to bleaching: Patterns of prevalence and severity

#### Bleaching prevalence

**Fig 2:**
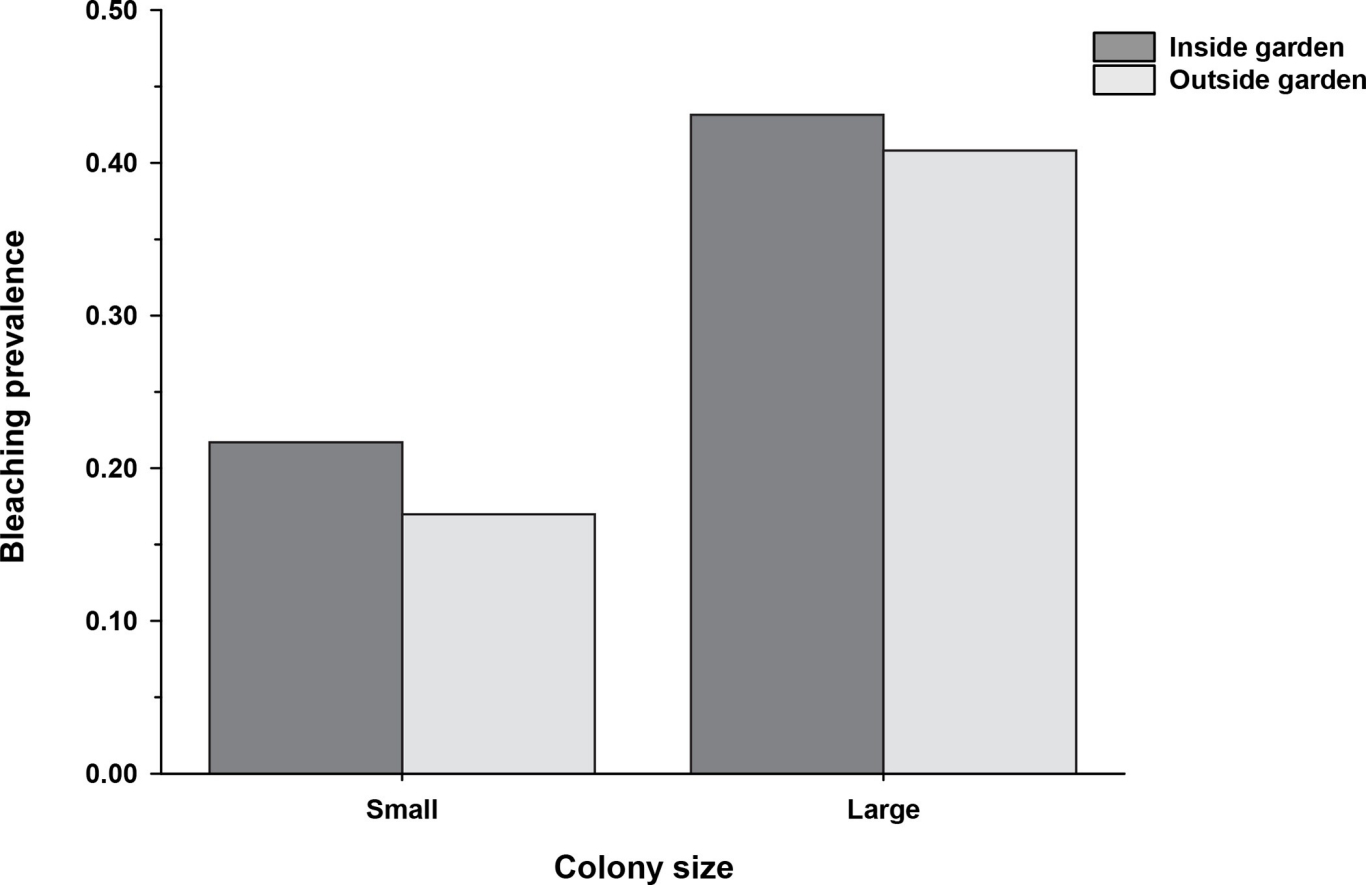
Bleaching prevalence as a function of farmerfish garden status. Bleaching prevalence (proportion of the sampled colonies that bleached) by colony size class as a function of farmerfish garden status for the 1,137 *Pocillopora* encountered on 178 bommies (number of bleached colonies: small: N = 105 inside, 53 outside; large: N = 126 inside, 20 outside; see Fig 1 for the total coral numbers by size and garden status).

Occurring in a garden did not have a statistically significant effect on the proportion of all small (*X*^2^_(1, 796)_ = 2.35, *p* = 0.12) or all large colonies (*X*^2^_(1, 341)_ = 0.02, *p* = 0.88) that bleached (i.e., bleaching prevalence) (Fig 2). Overall, a higher proportion of the total number of large colonies sampled bleached relative to smaller corals (*X*^2^_(1, 1137)_ = 63.1, *p* < 0.0001). Bleaching prevalence was about twice as great for large relative to small corals (Fig 2).

#### Bleaching severity

**Fig 3:**
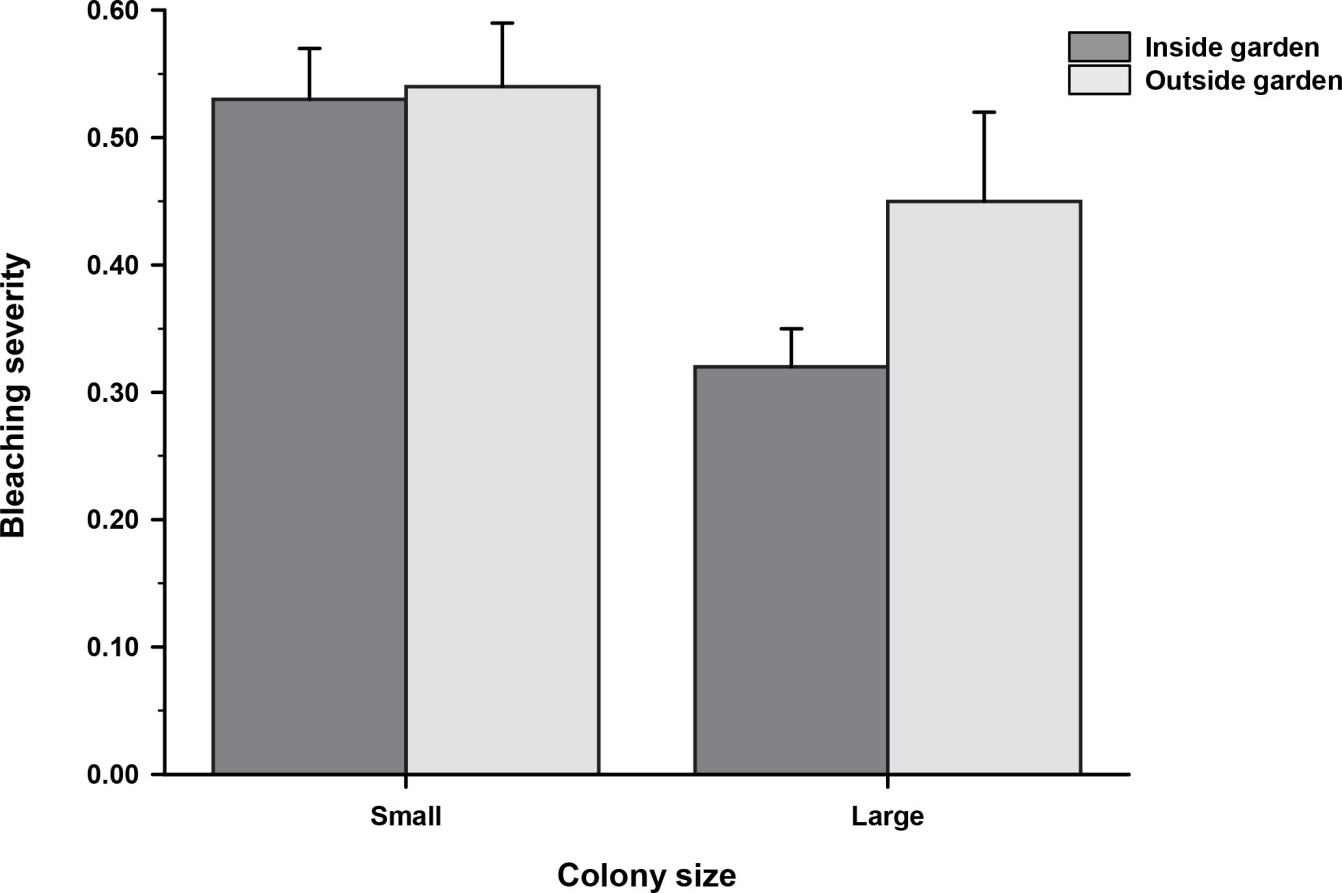
Bleaching severity as a function of farmerfish garden status. For colonies with ≥ 5% bleached tissue, the mean (± SE) bleaching severity (proportion of a colony’s tissue that bleached) by colony size class as a function of farmerfish garden status. Small colonies were more severely bleached (*p* < 0.005). See Fig 2 for the numbers of bleached colonies by size and garden status.

With respect to bleaching severity (i.e., the proportion of a colony’s tissue that bleached), the interaction between garden status and colony size was not significant (F_1, 203_ = 1.35, *p* = 0.24). Similar to prevalence, occurring in a garden did not alter how much of an individual colony’s tissue bleached, given that at least some tissue did (F_1, 203_ = 1.03, *p* = 0.31; reduced model without interaction term) (Fig 3). As with prevalence, colony size did have a significant effect on bleaching severity (Fig 3) (F_1, 203_ = 8.16, *p* < 0.005; reduced model without interaction term). Overall, small colonies had a mean severity that was roughly 40% greater than that of large colonies (Fig 3, Table 1). Taken together, these findings indicate that occurring in a farmerfish territory did not reduce or increase the probability of or degree to which *Pocillopora* tissue bleached in response to prolonged thermal stress.

**Table 1:** Bleaching severity of corals as a function of colony size and farmerfish garden status.

Colony size	Garden	No. of colonies bleached	Mean proportion of live tissue	SE	Bleaching severity	SE
Small	Inside	105	0.91	0.02	0.53	0.04
Small	Outside	53	0.98	0.01	0.54	0.05
Large	Inside	126	0.78	0.02	0.32	0.03
Large	Outside	20	0.79	0.06	0.45	0.07

The mean (± SE) proportion of live tissue of an individual coral colony and mean (± SE) bleaching severity (i.e., proportion of live tissue bleached) as a function of coral colony size class (small: ≤ 10 cm dia; large > 10 cm) and farmerfish garden status (inside vs outside); N = 1,137 *Pocillopora* colonies encountered on 178 bommies; corals were categorized as bleached if ≥ 5% of live tissue was bleached.

#### Fate of bleached coral: Patterns of mortality and recovery

The 399 bleached colonies followed for a year showed qualitatively identical patterns of bleaching severity as the larger collection of 1,137 colonies surveyed in June 2019 (Table 2). Bleaching severity was greater for small colonies, and within each size category, did not differ with garden status.

**Table 2:** Bleaching severity and patterns of recovery after one year as a function of colony size and farmerfish garden status.

Colony size	Garden	No. of colonies	Initial mean proportion alive	SE	Proportional change in live tissue after 1 year	SE	Initial mean bleaching severity	SE
Small	Inside	172	0.93	0.01	-0.63	0.03	0.43	0.03
Small	Outside	123	0.94	0.01	-0.72	0.04	0.45	0.03
Large	Inside	91	0.88	0.02	-0.12	0.03	0.24	0.02
Large	Outside	13	0.88	0.06	-0.26	0.1	0.29	0.07

The initial mean (± SE) proportion of live tissue, the proportional change in live tissue after 1 year, and the initial bleaching severity for 399 uniquely marked colonies followed for a year post bleaching as a function of coral colony size class (small: ≤ 10 cm dia; large > 10 cm) and farmerfish garden status (inside vs outside).

**Fig 4:**
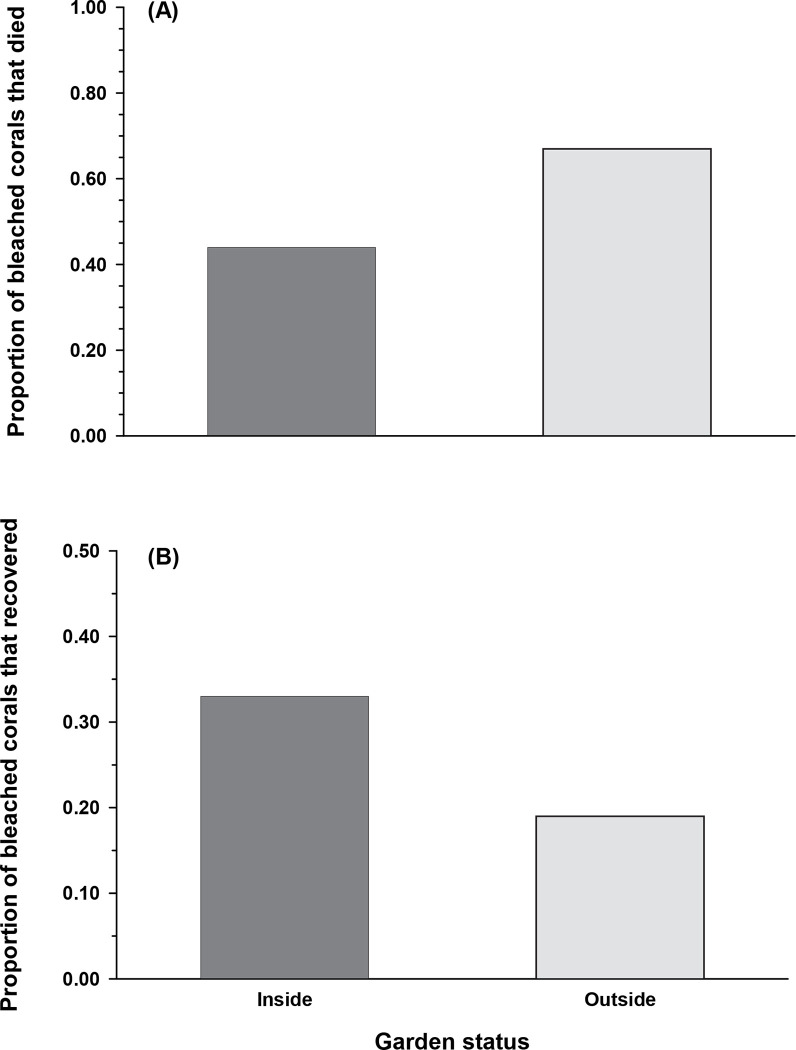
Fate of coral colonies after one year as a function of farmerfish garden status. (A) Proportion of the 399 bleached colonies that died after one year as a function of garden status. Colonies were considered dead if they lost all living tissue. (B) Proportion of bleached colonies that recovered after 1 year as a function of farmerfish garden status; a colony was deemed recovered if it had as much or more live tissue as it had in the initial post-bleaching survey.

*Complete colony death*. Garden status had a strong effect on whether a bleached colony completely died (*X*^2^_(1, 399)_ = 19.6, *p* < 0.0001). Just over 67% of bleached colonies outside of gardens did not survive the year compared to only 44% in gardens (Fig 4A). Thus, bleached colonies associated with farmerfish territories were about one third less likely to die. As expected, small colonies were more likely to die compared to large colonies (Table 3). Also as predicted, small colonies were less likely to die when they were associated with a farmerfish garden (*p* = 0.03; 1-tailed test). Large colonies exhibited the same, but non-significant (*p* = 0.36, Table 3) direction of change; the limited number of adult colonies likely prevented a robust evaluation of the relationship.

**Table 3:** State (alive or dead) of coral colonies after one year.

**Small colonies**					
Garden	Colony state		No. of colonies	Proportion of colonies by garden status	
Inside	Alive		65	0.38	
Inside	Dead		107	0.62	
Outside	Alive		33	0.27	
Outside	Dead		90	0.73	
**Large colonies**					
Garden	Colony state	No. of colonies			Proportion of colonies by garden status
Inside	Alive	83			0.91
Inside	Dead	8			0.09
Outside	Alive	11			0.85
Outside	Dead	2			0.15

The proportions of live and dead large colonies (‘colony state’) after one year as a function of colony size and farmerfish garden status.

### Colony recovery

Association with a farmerfish garden increased the chances that a bleached coral recovered by our operational definition (i.e., the proportional area of a colony covered by live tissue after a year was equal to or greater than at the time it bleached in 2019) (*X*^2^_(1, 399)_ = 7.53, *p* < 0.01). Using our threshold metric of recovery for colony sizes combined, 33% of bleached colonies recovered inside gardens (86 of 263) compared to just 19% of bleached, non-garden corals (26 of 136; Table 4, Fig 4B). This indicates that the ‘recovery’ advantage of dwelling in a farmerfish garden was almost double.

**Table 4:** Recovery of coral colonies after one year.

**Small colonies**				
Garden		Recovered	No. of colonies	Proportion of colonies by garden status
Inside		Yes	42	0.24
Inside		No	130	0.76
Outside		Yes	21	0.17
Outside		No	102	0.83
**Large colonies**				
Garden	Recovered		No. of colonies	Proportion of colonies by garden status
Inside	Yes		44	0.48
Inside	No		47	0.52
Outside	Yes		5	0.38
Outside	No		8	0.62

The proportion of surviving colonies that either did or did not recover to at least their initial amount of live tissue after a year as a function of colony size and farmerfish garden status.

## Discussion

Like many coral reefs worldwide, those surrounding Moorea recently experienced an intense thermal stress event that caused widespread bleaching and death of mainly branching corals [4, 30, 40]. For reefs in the lagoons of Moorea, mass mortality of coral can trigger a regime shift from coral to macroalgae that is challenging to reverse [41, 42], making it important to understand factors that enhance the resilience of coral to marine heat waves. Here we found that prevalence and severity of bleaching of *Pocillopora* colonies did not differ significantly inside or outside of defended gardens of the farmerfish *Stegastes nigricans*. Similarly, Smith et al. [43] found that branching coral under canopies of macroalgae (primarily *Sargassum* spp.) on fringing reefs of Magnetic Island on the Great Barrier Reef had the same susceptibility to bleaching as those in nearby plots where macroalgae had been removed well before the thermal event. However, with respect to post-bleaching recovery, we found that bleached *Pocillopora* colonies in a farmerfish garden had a substantially lower probability of completely dying (about one third less) and about twice the probability of recovering (having as much or more live tissue a year later) compared to their counterparts on open reef substrate outside of *Stegastes* territories. This rescue effect of dwelling in a farmerfish garden of filamentous algae stands in sharp contrast to the generally adverse effect that macroalgae have on the recovery of bleached coral [12, 43].

It is well known that despite the potential for some deleterious effects [44] the territorial gardens cultivated by *Stegastes nigricans* can enhance recruitment and growth of branching and other corals [10, 13–17]. This ultimately can result in a greater number and diversity of corals within a farmerfish garden relative to adjacent reef substrate of the same area [10, 13, 14]. While we also found a much greater density of individual *Pocillopora* colonies within gardens, our surveys also revealed that > 85% of the larger-sized colonies (i.e., > 10 cm dia) occurred in *Stegastes* territories despite that microhabitat accounting for < 40% of the reef substrate suitable for coral (i.e., surfaces with closely cropped turf algae or otherwise occupied by coral). This suggests that *Pocillopora* in farmerfish gardens grew faster, survived better, or both. We found that overall, bleached corals survived and recovered better if they occurred in a garden. As the frequency of thermal stress events continues to increase, the rescue effect of bleached coral in farmerfish gardens (lowered death rate, accelerated recovery of survivors) will likely further skew the distribution of large *Pocillopora* colonies towards *Stegastes* territories. Large colonies are the primary source of sexually produced propagules on reefs [4]. Further, Johnston et al. [45] found that smaller *Pocillopora* sp. colonies that recovered from bleaching suffered a prolonged period of reproductive impairment that was not observed in larger conspecifics. These aspects have profound population-level consequences that will further amplify the importance of farmerfish garden oases in enhancing the resilience of coral communities as the thermal stress regime continues to intensify.

The observed rescue effect of farmerfish territories on bleached *Pocillopora* was not just due to the difference in the colony size distributions between garden and non-garden habitats; small corals that bleached were significantly more likely to survive and recover if they occurred in a *Stegastes* garden. Because smaller colonies have lower energetic reserves than larger colonies [46], the underlying mechanism accounting for the enhanced survival and recovery of small *Pocillopora* in farmerfish territories may be related to how *Stegastes* interactions help bleached coral conserve energy needed for recovery and/or speed energy production. One possible mechanism is the protection corals in farmerfish gardens gain against corallivorous fishes, which *Stegastes* aggressively exclude from their garden [15]. Corallivory generally reduces colony growth and survivorship [47, 48], and several studies have shown that corals protected by farmerfish incurred substantially lower rates of corallivory that resulted in greater growth and survivorship than adjacent, unprotected colonies [10, 14]. Repair of wounds from corallivores and other agents of damage is a high priority for *Pocillopora* [45–49], and smaller colonies heal more slowly than larger conspecifics due to high energetic demand relative to available reserves [50]. Bleaching greatly reduces the already limited energy supply of a small coral such that colonies protected from corallivory by farmerfish may gain a fitness advantage by needing to devote less energy to wound repair.

Farmerfish interactions may enhance the production of energy by coral colonies via heterotrophic or autotrophic pathways. While the ambient flux of zooplankton appears sufficient to meet the basic heterotrophic needs of *Pocillopora* at our study location when colonies are not thermally stressed [51], it can become a crucially important source of energy when corals are severely bleached [52]. In the field experiments reported by Adam et al. [53] where reduced predation on *Pocillopora* colonies resulted in faster growth, the polyps of corals in lower predation treatments were consistently observed to be extended and feeding throughout the day, whereas diurnal feeding was not observed for corals exposed to ambient levels of predation. This suggests that thermally-stressed corals protected by farmerfish may be able to increase their nutrient intake by feeding on zooplankton without great risk during the day, and not just at night. Alternatively, local fertilization by nitrogenous excretions from damselfishes can enhance the growth rates of coral, including *Pocillopora* in Moorea [22, 23]. Fish excretion in the form of recycled ammonia and urea has been shown to increase the density of algal endosymbionts in bleached coral, which recovered faster and survived better than bleached colonies exposed to other forms of nitrogen [25]. While increases of nitrogen at low levels can be beneficial, an excess can become an added stressor to coral, thus increasing the potential for bleaching [11, 25, 29]. Existing evidence suggests that *Stegastes* in Moorea increase the supply of nutrients in their gardens that benefit algae. In laboratory experiments, growth of number of algal taxa in Moorea was enhanced with increasing nutrient concentrations [54, 55], while a field nutrient bioassay revealed that growth of algae in *Stegastes* gardens was greater when the farmerfishes were present than when experimentally excluded [39]. More research is needed to understand the mechanisms underlying potential positive effects of farmerfishes on post-bleaching recovery of small coral via heterotrophic or autotrophic pathways.

Marine heat wave events that induce mass coral bleaching will continue to occur more frequently and with greater intensity as the climate continues to warm [3, 9, 11]. As such, climate-related bleaching mortality of coral is now considered to be the one greatest threat to coral reefs in the foreseeable future [3, 9]. This places a premium on understanding factors that either moderate or exacerbate the impact of thermal stress on corals [25, 43]. Branching corals that recruit to defended territories of *Stegastes nigricans* are buffered to a greater degree against the most severe outcomes of thermal stress relative to colonies on adjacent substrate because smaller, more thermally-sensitive sizes are more likely to survive and recover, and because they are more likely to grow to larger, less sensitive sizes. The importance to coral resilience of this rescue effect of farmerfish oases will continue to grow as thermal-stress events become more frequent in the coming decades.
